# SUSTAINING VALUES AND CHARTING FUTURES

**DOI:** 10.1093/geroni/igae098.1652

**Published:** 2024-12-31

**Authors:** Kathleen Wilber

**Affiliations:** University of Southern California, Los Angeles, California, United States

## Abstract

In this presentation, Dr. Kathleen Wilber will lead a panel discussion exploring enduring values and evolving challenges within the field of gerontology, aiming to guide the trajectory of the discipline into the future. Dr. Wilber’s research is dedicated to enhancing health outcomes and quality of life for vulnerable older adults through systemic improvements in healthcare delivery. Her expertise in collaborative care models has contributed to understanding cost-effectiveness and optimizing service delivery structures. Recognized for her leadership in gerontology, Dr. Wilber was honored with the prestigious Donald P. Kent Award from the Gerontological Society of America (GSA) in 2023. In her presentation, she will share insights on timeless values that continue to shape the field and propose strategies for addressing persistent challenges. Dr. Wilber will also offer valuable advice for emerging scholars based on her personal journey, reflecting on lessons learned and potential areas for improvement. The panel discussion will further explore visions and directions for advancing the society. Following the moderated discussion, the audience will engage in an interactive question-and-answer session, fostering collaborative dialogue and actionable insights. By deliberating on enduring values, emerging challenges, and future directions, this presentation aims to catalyze concrete action plans to propel the society forward.

